# Cultural tightrope walkers: a qualitative study of being a young refugee in quest for identity and belonging in Norway

**DOI:** 10.1186/s40359-025-02484-8

**Published:** 2025-02-17

**Authors:** Per-Einar Binder, Hanna Heggen, Elisabeth Buer Vase, Gro Mjeldheim Sandal

**Affiliations:** 1 Department of Clinical Psychology, University of Bergen, Bergen, Norway; 2 Division of Mental Health and Addiction, Vestfold Hospital Trust, Tonsberg, Norway; 3 Administrative Research Institute, AFF, Norwegian School of Economics, Oslo, Norway; 4 Department of Psychosocial Science, University of Bergen, Bergen, Norway

**Keywords:** Refugee identity, Cultural adaptation, Acculturation challenges, Narrative identity, Sense of belonging, Bicultural integration, Young refugees in Norway

## Abstract

**Background:**

Refugee experiences significantly challenge personal identity, especially for youth orienting themselves in new cultural contexts. The study explores the complex process of balancing expectations from two cultures: how do formative encounters in Norway mold the self-perception and sense of belonging of those who arrived as child and adolescent refugees?

**Methods:**

A qualitative approach in which life story interviews were conducted with eight young refugees who arrived in Norway between the ages of 8 and 17 years was used. Through thematic analysis grounded in hermeneutical phenomenology, we analyzed their narratives to identify core experiences and perspectives. The interviews lasted 90–150 min, providing insights into personal identity development amid cultural adaptation.

**Results:**

A recurring and overarching theme is that participants grapple with feelings of “outsiderness” both in Norwegian society and within their culture of origin, creating a dual sense of alienation. This experience is painful, but they also perceive that it has given them the opportunity to develop open-mindedness and a unique perspective on cultural existence. Three subthemes were identified that describe various aspects of these narratives in greater detail: (1) Experiences of being a stranger; (2) value conflicts, but value reconciliation is possible; and (3) a unique perspective: navigating between two cultures. The narratives highlight the complex negotiation between maintaining heritage and adapting to Norwegian norms.

**Conclusions:**

The study of participants’ narrative identities reveals ways of coping with these transitions, revealing how participants continuously reconstruct their self-narratives to adapt to their new cultural environment. The findings suggest that while acculturation is a challenging process characterized by psychological tension and existential vulnerability, it can also foster personal resilience and new opportunities for meaning-making. The research contributes to understanding the refugee experience in Norway, emphasizing the need for social support systems that acknowledge both the struggles and potential for growth inherent in cultural integration.

## Introduction

Refugee experiences undoubtedly test a person’s identity. Fleeing one’s home country necessitates both recreating one’s self-narrative and establishing continuity with what has been [1]. Being a refugee involves more than physically relocating from one place to another—it affects all aspects of a person’s life [2]. The experience of fleeing is seen as a critical disruption in people’s lives that impacts the construction of personal autobiographies and identities [3].

In this study, we understand personal identity as an individual’s self-definition as expressed through narratives, core value choices, relationships, and roles that are important for self-perception [4]. Moving from one country to another can lead people to experience discontinuity in their personal identity [4, 5]. The refugee experience requires recreating one’s life story by making meaning of what one has been through [1].

Identity is not static. Self-definitions and self-perceptions are constantly developing and being constructed as life changes. Sociologist Anthony Giddens [6] writes that “A person’s identity is not found in behavior or - in others’ reactions, but in the ability to keep a particular narrative going” (p. 54). This underscores the importance of an individual’s subjective story in shaping their personal identity. The experience of being a refugee puts the ability to keep the story going to the test.

McAdams strongly emphasizes coherence in life experience, partly for good reason; as we have seen, the ability to create coherence has been linked to several positive health benefits. At the same time, life is more than and different from storytelling. As Bruner [7] noted, our here-and-now experiences are complex and not infrequently chaotic. The life story is not a finished product. It is something that “we construct and reconstruct constantly to meet the needs of the situations we encounter, and we do this with the help of our memories of the past and our hopes and fears for the future” [7, p. 210]. Both the stories we tell and the stories told about us are constantly changing and evolving as our lives change: “our stories about ourselves must fit new circumstances, new friends, and new enterprises” [7, p. 210]. Migration, especially being a refugee, is a dramatic example of entering new circumstances.

Working with life stories in a refugee context offers a range of possibilities. The narrative approach enables consequential and relatively direct communication of refugees’ voices. Through their own words and stories, we can capture the uniqueness, complexity, and richness of each refugee’s experiences [8].

Numerous studies have focused on risk factors associated with being a refugee, such as long-term stress and psychological impairment in refugee experiences [9–11]. Refugees are at risk of experiencing traumatic events before, during, and after fleeing [11–13]. They are forced to leave their homes, families, and cultures. At the same time, they must find their place in a new society and community. A lack of connection and familiarity with the new culture can lead to isolation and feelings of alienation, affecting refugees’ health [14, 15].

Acculturation has significant implications for the mental health and overall well-being of refugees and migrants. As Lackland Sam and Berry [16] highlight, the strategies individuals employ to navigate their new cultural environment can have profound effects on their psychological adjustment and social integration.

The four acculturation strategies described by Sam and Berry—integration, assimilation, separation, and marginalization—represent different approaches to balancing one’s heritage culture with the host culture. Integration, which involves maintaining aspects of one’s original culture while also adopting elements of the new culture, is generally associated with the most positive mental health outcomes [17]. This strategy allows individuals to retain a sense of cultural continuity while also developing the skills and connections necessary to thrive in their new environment.

In contrast, marginalization, where individuals distance themselves from both their heritage culture and the host culture, is consistently linked to poorer mental health outcomes, including higher rates of depression, anxiety, and social isolation [18]. This underscores the importance of cultural connection and social support in acculturation.

The central question of “How much shall I change?” is at the heart of the acculturation process. As Schwartz et al. [19] noted, the clash between collectivistic and individualistic cultural norms can be a significant source of stress and conflict for refugees and migrants coming from more traditional societies to Western countries. Likewise, those from hierarchical cultures might find it challenging to accommodate more egalitarian societies. Cultural distance can make the integration process particularly challenging, as individuals must handle and reconcile potentially conflicting value systems and social expectations. The questions that often arise are “How much should I retain norms and traditions from my culture of origin?” and “How much should I absorb the norms and traditions of the culture of my ‘new’ home country”?

Age plays a crucial role in the acculturation process, with younger individuals generally showing greater flexibility and adaptability in adopting new cultural norms and practices [20, 21]. This is partly due to the ongoing process of identity formation in youth, which allows for easier incorporation of new cultural elements. However, this generational difference can also lead to intergenerational conflicts within refugee families, as younger members may acculturate more rapidly than their elders do.

However, acculturation is not a linear process, and individuals may employ different strategies in different domains of their lives or at various times. Factors such as the receptiveness of the host society, the presence of coethnic communities, and individual personality traits all play roles in shaping acculturation trajectories [22].

Furthermore, the stress associated with the acculturation process, often referred to as acculturative stress, can have significant impacts on mental health. This stress can be exacerbated by factors such as discrimination, language barriers, and socioeconomic challenges. Addressing these stressors and providing appropriate support systems are crucial for promoting positive acculturation outcomes and mental health among refugee populations [23].

Many refugees experience trauma burdens that impact their quality of life and adaptation [24]. However, we also see many who find ways to relate to painful experiences that open up opportunities to discover new values and make enriching reprioritizations in life [9].

Many aspects of having gone through a refugee experience challenge one’s sense of meaning and purpose. Refugees often suffer great losses that disrupt or prevent them from pursuing their life goals, thus depriving them of much of what previously gave life meaning [25]. Some studies of refugees have shown that seeking and finding meaning is a process in which strategies that provide adaptation at one point may hinder adaptation at a later point [26]. In a refugee camp, for example, it may work best to focus on the here and now and take one day at a time [27] When beginning a new life in a new country, one needs to open up to a much larger time horizon. One needs to be able to choose life goals adapted to having a predictable future. One must address contradictions between what has been and what is now, which—when things can go well—can provide new direction and meaning in life. Being able to make a positive difference for others even during the waiting period for asylum will be an important first step for many in regaining a sense of purpose and meaning [27, 28]. Starting anew in a new culture can provide growth opportunities. Previous studies have revealed several potential growth-promoting factors related to migration and postflight experiences [29–32].

In this study, we have based our approach on McAdams’ life story interview (35, adapting it with questions related to coming to Norway as a refugee at a young age, specifically the 8–17 age group). This involves coming to Norway at an age where one is old enough to have episodic memories while still young enough to learn the deep structure of Norwegian grammar.

Our ability to tell stories is connected to one of our existential basic conditions as human beings do. We are meaning-making creatures. Stories are one of the foundations of our identity [34]. The self-narrative is intertwined with how we both define ourselves and are defined through the relationships we are part of. They are connected to the values we believe in, commit to, and base our actions and choices on. Moreover, life stories weave into our bodily and emotional way of being in the world.

Stories, relationships, values, and bodily being can be seen as four fundamental—yet unstable—areas where we enact and build our identities [4]. These areas must be nurtured and cultivated. Identity is inextricably linked to existential vulnerability. Life stories are ambiguous and changeable; they take unexpected paths. In our relationships, we can, at any time, be defined in new ways. Our values are based on the choices we have made. When life-changing events occur, we may choose different options. The body is constantly changing. Our emotional experiences are bodily, and they are changeable. At times, experiences become painful and distressing; this is also an inescapable condition of being human [35]. Our bodily being opens up the possibility for unfolding in the world. At the same time, our attachment to the body reminds us that we will die one day.

We can distinguish between vulnerability related to specific life conditions and existential vulnerability [4, 36]. War, flight, and emotional pain and trauma are specific and historically conditioned vulnerabilities. Moreover, such upheavals in real life make universal and existentially conditioned vulnerabilities clear and necessary to relate to. In analyzing these life story interviews, we explore the intersection between the historically conditioned experience of flight and finding belonging in a new homeland, on the one hand, and understand these experiences in light of the existential basic conditions that humans primarily have in common.

This study aims to uncover the pivotal experiences that shape the identities of young refugees as they navigate life in Norwegian society. More specifically, we explore the following: How do formative encounters in Norway mold the self-perception and sense of belonging of those who arrived as child and adolescent refugees?

## Materials and methods

### Participants

Eight individuals (four women and four men) were recruited for our study. The youngest participant was 21 years old, whereas the oldest was in the middle-40 s. The participants had arrived in Norway between the ages of 8 and 17 years and had lived in the country for 13–30 years (Table 1). All participants were assigned pseudonyms.

**Table 1:** Demographic and Migration Characteristics of Study Participants

Pseudonym	Age at Arrival	Current Age	Region of origin
Nora	16	33	Central Africa
Sara	17	40	Middle East
Marlon	16	36	Middle East
Amina	16	35	Middle East
Leyla	12	35	Middle East
Vince	10	45	South East Asia
Razm	10	25	Central Asia
Samira	8	21	East Africa

During the recruitment period, we aimed to connect with individuals who had fled to Norway at a young age and were over 21 years old at the time of the study. Some participants were recruited through an organization associated with the business community in Bergen. A standardized message, which provided information about the main themes of the study and the participant criteria, was shared on various social media platforms. We subsequently employed the snowball sampling method, allowing those we initially contacted to explore their own networks for potential participants.

After establishing contact with suitable participants, an email was sent containing detailed information about the project, along with a consent form outlining anonymization procedures and participants’ rights.

### Procedure

Five of the interviews were conducted digitally via Zoom, and three interviews were conducted physically in locations where privacy was safeguarded. The interviews lasted from 90 to 150 min. The participants varied in their storytelling style, rhythm, and verbal fluency. We, therefore, allowed the interviews to last as long as each participant felt needed to explore their life stories. We invited the participants to a short conversation approximately two weeks after the main interview. Five such conversations were held, as three of the participants did not want a conversation. The aim of this meeting was to get to grips with the thoughts and feelings that the participants were subsequently left with.

The interviews aimed to better understand how persons who fled to Norway at a young age constructed their narrative identity and, through this, how they related to and reflected upon their lived experiences.

The first author conducted two of the interviews, the second author four, the third author three, and the last author one.

The original English version of the life story interview is available online (McAdams, 2008). The first author translated this interview guide into Norwegian. A native English speaker who also speaks Norwegian fluently confirmed that the translation captured all the meaning.

The interviewer began by asking the interviewees to provide an overview of their lives, conceptualizing their life as if it were a book or novel and dividing it into “chapters” with a brief plot summary for each. The interviewees were subsequently asked to focus on a few key events that were particularly memorable or important in their life stories. These events included a high point, a low point, a turning point, a high point in childhood, and a low point in childhood, along with other scenes that were notable for their emotional significance or meaning for their life course. For each scene, the interviewees were asked to describe when and what happened at that moment, who was present, what they were thinking and feeling, and how the scene was ultimately resolved. Additionally, they were prompted to reflect on what they believe the scene reveals about them as a person and the insights about their life story that might be derived from the scene. We have added two questions that were not included in McAdams’ [33] original version: The first question explores how coming to Norway as a refugee might affect a person’s beliefs and values. The second question explores how belonging to two cultures might affect one’s beliefs and values.

### Data analysis

A qualitative method was employed to preserve the thematic content of the interviews, allowing for the exploration of nuanced meaning-making in participants’ life stories [37]. This explorative-reflexive thematic analysis was grounded in hermeneutical phenomenology as an epistemological framework [38]. The approach emphasized exploring participants’ concrete lived experiences while acknowledging their symbolically mediated prenarrative quality [39].

In alignment with the hermeneutic ideal of the reflexive methodology, the researchers engaged in dialog with participants’ narratives to heighten awareness of their own preconceptions and reflect on the interpretive lenses shaped by their professional and theoretical backgrounds. This reflexive stance aimed to address potential biases in both interviews and transcript analysis [40, 41].

The analysis proceeded through six steps:

1. All the researchers read the transcribed material to gain a comprehensive understanding of each participant’s life story.
2. The first author identified thematic units relevant to the research question.
3. The first author developed “meaning codes” for these units to facilitate later retrieval (Kvale & Brinkmann, 2009).
4. The first author grouped text segments according to these codes.
5. The first author interpreted and summarized the coded text groups into meaning patterns and themes reflecting participants’ formative encounters in Norway that shaped their self-perception and sense of belonging.
6. All the authors reviewed the overall text to ensure a comprehensive representation of voices and perspectives, with the fourth author critically auditing the identification of thematic units and meaning patterns.

“Meaning patterns” emerged from condensing and comparing relevant meaning units across participants’ narratives (Binder et al., 2012). A pattern was identified when there was convergence between different participants’ narratives with moderate divergence, resulting in thematic richness. Themes were formulated to express the narrative content of these meaning patterns, combining hermeneutic interpretation with phenomenological commitment to participants’ lived experiences.

An example of this process can be seen in the theme *There will be value conflicts*,* but value reconciliation is possible*. The term ‘value’ is an interpretive construct derived from the analysis of participant narratives. For instance, Sara described ambivalence regarding strict cultural norms, which we interpret as reflecting both a longing for protection and a desire for individual autonomy. Similarly, we see Vince’s struggle to balance parental expectations with his athletic aspirations as a conflict between familial duty and personal ambition. These experiences initially appeared as individual motivational conflicts, and we still think they should rightly be regarded as such at an individual level. However, through comparative analysis across multiple participants’ narratives, they also revealed a broader pattern: the negotiation of values under cultural and social pressures. This interpretive process involved moving between participants’ lived experiences and an overarching hermeneutic framework to identify the thematic structure.

One example of how we “engaged in dialogue with participants’ narratives” can also be found in our analysis of cultural and personal conflicts, such as wearing the hijab, dating, or pursuing athletics. We initially engaged empathetically with participants’ accounts, particularly regarding the emotional weight of these conflicts—acknowledging the pain, uncertainty, and relational struggles they described. However, we also actively explored how participants themselves reflected on these experiences in terms of values, identity, and broader societal discourses. For instance, discussions about the hijab frequently carried multiple layers of meaning: personal autonomy, family expectations, religious identity, and political debates on integration in Norway.

While we shared a common thematic formulation, our research team contributed with different interpretive angles. Some emphasized the existential and identity-related tensions participants faced, others focused on the intersection between personal narratives and societal structures. This diversity in perspectives helped us recognize the nuances in how participants positioned themselves: for example, the distinction between critiques of the hijab from within the culture—voiced by participants navigating their personal and religious beliefs—versus critiques rooted in an integration perspective, and, more starkly, the rejection of hijab by certain anti-Muslim groups. By engaging with these complexities, we sought to avoid presenting participants’ narratives as overly “neat” or reducing them to pre-existing political or ideological frameworks.

This approach acknowledges the interpretive nature of narratives and views the study of narratives as an interactional process where findings result from the interplay between researchers and participants.

### Researchers

The study was conducted within the Society and Workplace Diversity Group. The first author is a professor of clinical psychology with 29 years of clinical experience, broad experience with qualitative research methods, and an interest in existential approaches within theoretical and philosophical psychology. The second author is a psychologist currently working at the Administrative Research Institute, AFF, at the Norwegian School of Economics. The third author is a psychologist currently working in an outpatient clinic for patients with psychosis. The fourth author is a professor of personality psychology and has broad experience with research on refugees’ mental health and integration into the society of settlement.

### Ethics approval and consent to participate

All participants provided written informed consent in accordance with the Declaration of Helsinki (World Medical Association, 2013). The interviews were audio recorded and stored securely on the SAFE (Secure Access to Research Data and E-infrastructure; University of Bergen IT Desk, 2019) platform, adhering to the Norwegian code of conduct for information security in the healthcare sector.

While it is standard to seek consent from legally authorized representatives (LAR) for vulnerable populations such as refugees, our study participants, although they arrived in Norway as refugees, are now adult Norwegian citizens with full decision-making capacity. They are well-integrated into society and were fully capable of understanding the study’s purpose, implications, and procedures. The Norwegian Centre for Research Data (NSD) approved our consent procedures, confirming that direct informed consent from the participants themselves was both appropriate and sufficient for this research.

Participants received detailed information about the study in advance, including the consent forms, and were encouraged to ask questions at any point. To protect their privacy, pseudonyms were assigned to all participants, and identifying information was modified to ensure anonymity.

### Findings

In the analysis of these life stories, a recurring and overarching theme is that establishing a personal identity and sense of belonging involves actively finding ways to relate to contrasts and differences in norms and values between the groups to which they belong and often having to manage experiences of outsiderhood both in relation to the majority culture and their own. This experience is painful, but they also perceive that it has given them the opportunity to develop open-mindedness and a unique perspective on cultural existence. Three subthemes were identified that describe various aspects of these narratives in greater detail: **(1)** Experiences of being a stranger; **(2)** value conflicts, but value reconciliation is possible; and **(3)** a unique perspective: navigating between two cultures.

### Experiences of being a stranger: outsiderness among those I came to and those I left behind

The experience of otherness in Norwegian society is a recurring theme in all participants’ narratives, ranging from a central focus to a secondary issue. This feeling is described as most intense upon arrival but persists to varying degrees throughout their stories. For some, the sense of outsiderness is tied to skin color and language, whereas for others, it stems from the disruption of taken-for-granted norms and interpretations from their country of origin. As participants gradually adapt to the rhythms and structures of Norwegian society, they establish a new sense of familiarity. However, a lingering sense of foreignness often remains.

The feeling of being outside the community of “Norwegianness” can persist over time. Simultaneously, some participants gradually experience a sense of disconnection from their culture of origin.

For Nora, today 33, who fled from a Central African country when she was 16, skin color became a central element in her feeling of difference: “When I came here, things went better than expected. It was a bit strange to start thinking about being brown in terms of identity. I knew it, of course. It was just odd to relate to it.”

For a Norwegian teenage girl, makeup and hair products are important. For Nora, these became confirmations of being different:

When I was supposed to hang out with the other teenagers, it was just very strange. I can’t use the makeup they use. They use this brown foundation, but it’s a bit too light for me, so I look like a ghost. I can’t use their hair products. I can’t read about it anywhere either. If you read those magazines, there were just white women there. They looked nice, but I can’t do what they do if I want to look nice too.

Language also confirmed a sense of outsiderness, especially initially: “People talked, and I heard nothing. I remember teachers coming into the class and telling good jokes, and the whole class could laugh except me. Because I do not understand what the teacher was talking about.”

Sara, today 40 years old, came to Norway when she was 17. For Sara, who became a young mother in her late teens, gaining acceptance was not easy: “When I picture myself as a youth, I see myself standing and knocking on an enormous door that will not open.” Sara describes her experience as a “limbo” state, where she is neither fully Norwegian nor truly connected to her country of origin:

I don’t belong to any culture; it’s really a limbo state… I don’t have contact with the ‘old’ environment. It’s too hectic for me. No, actually, I want to be left alone, and yes, I also need socialization. But this is an environment where when you come, it’s always complaining. I’m a bit past that phase of being from there and feeling belonging… I might listen to a song from my home country a couple of times. However, it is not like I sit and mourn over it; I’ve acknowledged that the lack is there. If I had moved back to my relatives now, I’m not sure it would have felt so good. I’m used to not relating to or answering anything regarding family. I very much control my own activity and my free time. Yes, I feel like I’m in between. Maybe more Norwegian, actually.

For Sara, her country of origin represents interpersonal “closeness.” Norway represents more freedom of choice but also greater interpersonal distance. When she steps out of her closer community, relationships and contact become something she constantly has to work for. In addition, she feels ambivalent. She appreciates freedom in Norwegian society, as well as the possibility of distance. However, the feeling of alienation often results in loneliness.

This is also the case for Samira, now 21. She was just eight years old when she arrived in Norway with her mother. As they moved frequently from place to place, she struggled to form connections with her peers. The pain of being different lingered in her memories: “I barely had time to settle in, and the other children were hostile… I can still picture them damaging my books. I remember feeling so alone when I first came here. Looking back now, it all seems clearer. When I joined sports teams, I was the only non-Norwegian child,” she recalls, her voice cracking with emotion. “I felt like such an outsider”.

Several participants described this experience of alienation from one’s own background. When Marlon visits his country of origin after fleeing, he realizes that he cannot control whether he has a sense of belonging to his old community: “I visited my country of origin once, don’t remember when it was. And the taxi driver asked me 100 times, ‘Where are you from?’ I just said, ‘I live in the city.’ He refused to believe it…” Marlon’s experiences are something several participants can identify with - falling between two stools or experiencing a lack of steady cultural belonging.

### There will be value conflicts, but value reconciliation is possible

Several participants described conflicts between values and norms in their countries of origin and Norway. These often revolve around the freedom to choose one’s own path and break with traditional norms and expectations. The participants expressed challenges and conflicts related to wearing the hijab, family expectations, and individual rights. These conflicts are often tied to gender roles, particularly for women. Many participants experience tension between the desire for personal freedom to choose relationships and affiliations and the need to respect and preserve relationships with their families and cultural backgrounds. This freedom, although often at great personal cost, provides opportunities to explore and develop their own identities and values in a society that values individual autonomy.

A key liberation for several female participants has been choosing not to wear the hijab. In their life stories, this only became possible when they fled their countries of origin. When discussing religious traditions, participants seem to want them to be reconciled with Western ideals of freedom, with the hijab taking on a special place in the struggle for self-determination. However, there is much more behind the freedom to dress as one wishes; it is about experiencing belonging to and acceptance from the community.

Leyla, who came to Norway at age 12 and is 35 today, has distanced herself from wearing the hijab: “I… I hated wearing the hijab, for example. It’s the worst thing to have to do” (Leyla). The free choice to wear the hijab is important to her, such that she wants to be with a man who respects that this is up to her and not a given based on culture. Amina describes the strong pressures to wear the hijab in her country of origin: “If you don’t do it, if you don’t cover your hair, then you’re the one who is different and stands out in a negative way.” A highlight from Sara’s childhood was the moment she took off the hijab and felt free: “Everything was so exciting, and taking off the scarf was almost like flying on clouds—it was so soft”.

Amina came to Norway when she was 16 and is 35 today. Amina further related that she always knew she wanted to take it off but that it has been challenging in the face of resistance from her family: “I still wanted to have contact with the family. Therefore, I used six years, the first six years in Norway, trying to talk to them - negotiate back and forth about not covering my hair.”. In the middle of the process, she received an ultimatum that put her integrity at stake: “The choice was… If you take off the hijab, you’re no longer our daughter”.

Amina initially had a good relationship with her father, who was also a role model for her growing up, and she described him as relatively liberal on religious and cultural issues. However, there was a major conflict between them when he demanded that she wear the hijab, and she refused. Then, he hit her, something he had never done before. This led to a change in her relationship with her parents. For a period, they had no contact but eventually managed to re-establish the relationship. However, the incident set in motion a larger change within her around questions of religion and faith:

We had a very good relationship with him and a lot of respect for him. They did something that made me start to see him as a human and question him: Is what he’s doing right? Is it wrong? So, I brought him down to a human level. But also, at that time the religious question came up, and it came because of covering the hair, so I’m a bit like… I was a child who thought a lot. And actually, I was quite spiritual, but if I hadn’t been hit that day, I might never have questioned whether God exists or not. It wouldn’t have been important to me, but for me it became a huge question. Does God really want me to belong and go to hell if I don’t do it?

For Amina, the conflict around the hijab and traditional Muslim norms is not primarily a conflict between the norms of her homeland and Norwegian norms. For some, the rebellion against the hijab began even before coming to Norway: “It’s not Norway. I’ve always known I wanted to”. At the same time, she points out that in her homeland, making such a choice would have major negative consequences.

By negotiating with her family and finally gaining acceptance for not wearing the hijab, she experiences both personal integrity and a preserved family relationship. This allows her to maintain important family connections while living in accordance with her own convictions.

Leyla has experienced strong directives on who she should and can be:

You are born into it, but still, you don’t quite fit in… I’ve grown up with everything being ‘no no no, you shouldn’t talk like that, you shouldn’t read like that, don’t sit like that’. Everything has been controlled in everyday life. It’s very deep inside me.

Leyla describes feeling quite unsure of who she is and says this means she “doesn’t really know what values and what boundaries I actually have - And can be very gullible and naive. So, it’s very, very difficult”. She has had some longer relationships with men, most of which she regrets. She also had a series of more casual short-term relationships with men during a period when she experienced major emotional difficulties. She now finds this difficult in terms of establishing a partner relationship because of the differences in norms between the Western dating culture she has been part of in recent years and the norms for relationships in her culture of origin. She has primarily had non-Norwegian friends. Until now, it has not been relevant for her to have a Norwegian boyfriend. At the same time, she feels that a European man has now become more relevant to her because she has become more distanced from her original culture:

I just fell in love with foreign boys. All the way up until after I made so many wrong choices where I slept with very many different ones. Then it was like okay, but I can’t be with a Muslim man. They can’t accept that I’ve slept with many. That has also made me think that then I’m not part of the culture in my country of origin. So, I’ve kind of thought that maybe I want a Norwegian or at least a European man.

Here, the condemnation that she anticipates from countrymen also affects her sense of identity. The potential condemnation weakened her sense of belonging to her homeland. In a way, she has already chosen European values, without first being aware that was what she was doing. At the same time, she still experiences a challenge in relation to expectations from home. Leyla is from a culture where the extended family’s opinions weigh heavily, further reinforcing her uncertainty: “Mom is more conservative, and she’s the one who’s going to struggle, for example, if I get a Norwegian man.”

Vince came to Norway when he was 10 and is 45 today. In Vince’s upbringing, there was a period where he experienced tension between respecting his parents’ expectations and realizing his own dreams in sports. Coming from a culture where respect for elders is a core value, Vince felt obligated to sacrifice his own desires if his parents set restrictions. Vince recounts that his team coach struggled to understand how he could allow his parents to limit his growing athletic talent, arguing that it was Vince’s life. For a time, Vince had to navigate conflicting views from adult role models regarding his freedom to choose. When Vince refused to contradict his parents’ opinions, the coach visited the family to reach an agreement. Compared with other participants’ experiences, Vince’s parents seemed more open to adapting to Western ideals of freedom. This allowed Vince to pursue athletic aspirations without compromising family expectations. This reconciliation provided him with a broader range of opportunities in various aspects of life.

Sara, as a first-generation immigrant and mother of two generations, describes raising children with freedoms different from her own. She characterizes her country of origin as closed, where concepts such as women’s liberation were not taught. Sara reflected, “We didn’t think; it was in a way reassuring and protective. When I see my son having challenges, we didn’t have choices; we had to accept what we had [our bodies]… so that confusion, it was more of a search outside oneself”. Growing up, Sara experienced a more predetermined path for one’s future. Accepting her children’s liberal attitudes towards identity has been a turning point for Sara:

(…) when your children come and have internalized Norwegian thinking, and they look down on you because you don’t have as many resources, you don’t have as much status. That’s the cultural struggle that’s really going on and the loneliness it brings.

While encountering new ideals of freedom has been costly for many, it is also praised. The participants described gaining new opportunities; freedom to break from religion; and choices in education, work, partners, and residence. Vince admires ideals in Norwegian society, and today, both he and his family have more educational and career opportunities. He proudly stated, “We are doing well. They (the children) will also grow up in a society where they have rights. That means everything; no one should dump us”.

The encounter with greater freedom has created space for many participants’ value systems to become more flexible. Razm,” who arrived in Norway at age ten from Central Asia and is 25 today, described that growing up in a community with much freedom has made him more open:

But for my part, I’m quite sure that if I had grown up in another country, I would have had completely different types of thoughts and values. But what I’ve noticed about Norway is that anywhere else in the world, the surroundings might have actively tried to influence me more to become like them, but in Norway, I’ve learned to get the freedom to think for myself, and through that, find my own way.”

Reconciling values from different cultural communities is a challenging and painful process. When participants manage to reconcile different values, they find that their lives are enriched with a broader perspective. This can lead to greater personal satisfaction, improved relationships, and more opportunities both socially and professionally.

### A unique perspective: navigating between two cultures

The participants who came to Norway at a young age were exposed to two cultures early in life. This contrast has, at times, felt like an exhausting split of self. However, participants described in various ways how these experiences of living in two different cultures and fleeing from one country to another at such a young age have had a significant effect on how they think about people, the system in Norway, and their perspectives on life. Growing up in two cultures has had major implications for the core values important to participants, including being adaptable, hardworking, open, respectful, and working for the well-being of all people. In their upbringing, they have been exposed to different worldviews, both religious and cultural, and consequently have developed respect and openness for differences. Participants wish to adapt and include both cultures in their daily lives, which involves significant shifts in value bases over relatively short periods. In other words, participants seem to have developed flexibility in handling everyday life.

The participants come from very different cultures, but a common denominator is that these cultures have a closer view of issues that Norway has a freer attitude toward, such as gender, faith, and sexuality. Several participants reported that they have distanced themselves from their family’s religious approach but that there are some safe frameworks within their culture of origin. They relate to these contrasts in everyday life, respecting and adapting to the differences.

Sara comes from a culture where the frameworks were strict and defined. She states, “We were not very enlightened. It was a rather closed environment. I hadn’t heard about women’s liberation. It wasn’t that I lived very oppressed. It was, in a way, quite protective.”

The Western opportunity to make choices gradually became increasingly important for Sara. She established herself with a Norwegian man and eventually made an independent choice of education. At the same time, she is critical of the strong focus on identity and identity-defining projects that have now become important for many youth in Western societies, especially in connection with her daughter being involved in such a project. She sees how groups of Norwegian youth become very preoccupied with defining who they are, preferably on the basis of something that is “inside” them. This contrasts with how she herself was searching as a teenager: “I wanted to become an astronaut or a doctor, that was the dream… it was more of a search outside myself, then.”

Amina, who, like Sara, came to Norway in her middle teens, has made clear choices against religious norms in Islam, which has also resulted in major conflicts with her parents. At the same time, she experiences an emotional connection to her original language. For her, it is as if her homeland’s language represents emotions, whereas Norwegian represents rationality:

I have feelings and memories from my homeland, but here is more my head, and I notice it in that I like to read textbooks in Norwegian. But I like to read fiction in Arabic. Because in Arabic, it’s a bit like the language I feel, but maybe don’t think. I think maybe I think about it to some extent, but if I want to be logical, or if I want to decide on something and make a decision or want to discuss something, it has to be in Norwegian. So, subjects are Norwegian, but the heart is Arabic.

While Amina has particularly experienced gender norms in Muslim culture as oppressive and life-limiting, she still has a strong emotional attachment to traditions:

I mean, I’m not religious at all, and I’ve been angry or not angry, I’ve had such painful feelings about religion for several years, but it’s been so long now that it… doesn’t bother me that I have this background, even though I don’t believe in God, but I call myself a cultural Muslim. I’m very fond of many - of several of the holidays, I like Eid.

Furthermore, Amina reflects on how she adapts to her family. It is especially important for her to have a relationship with her mother despite different cultural views. For Amina, belonging to two cultures is also experienced as a privilege: “I don’t identify with all Norwegian values either. It’s a bit like, I feel, that I respect both, and I have from both, but feel that I can choose, and then I feel privileged.”

Marlon, like Amina, has distanced himself from the religion he was born into. Marlon came to Norway alone at age 16 after a dramatic escape and is now 36. He experiences faith as supportive if he, for example, goes through something difficult, and therefore, it is sometimes nice to include it in everyday life:

I was born a Muslim, but I’m not religious. But when we humans are stuck, we pray to something, right? Either nature or God, yes, we ask for help. And I do that. It happens that I go to some events in the Mosque. However, other than that, I don’t think more about religion.

Leyla has had challenges with the conservative culture from which she comes. Her upbringing was characterized by strict rules and rituals that created challenges for her:

I struggle a lot and have had a very big identity crisis all my life from my teenage years, maybe even before. It has been that the actual culture of the country of origin, then, and that way of thinking, society, values have been… have not fit with me.

Despite the conservative culture she comes from, Leyla expresses that “the other side”, with its “everything is okay” attitude, can also become extreme. For her, it has been important to find a middle ground where like Amina and Marlon, she has a desire to integrate both cultures into her daily life. She particularly likes music, clothes, and some traditions. She describes that she includes elements from both cultures in her daily life and that an important value for her has been to show love for one’s neighbor rather than having strong religious and cultural affiliation.

Among Vince’s core values is being humble and adapting to the culture one comes to, and he believes it is a given and the least he can do since he has been given the opportunity to come to Norway:

If you, for example, invite me home, and I come to your house, the first thing I notice is how you do things at home. And I behave as you wish. It’s not like I go in and do exactly as I do at my home. I do it so that if you put the shoes there, I put my shoes there, if you stand, I stand, and if you say please sit, I sit down [laughter].

The value of being generous in meeting other people has created direction in Vince’s life. He described his adaptability as central to finding belonging and building relationships in Norway.

The fact that participants came to Norway at an early age, started school, and began learning the language relatively quickly is important for their sense of belonging to the Norwegian system. Schools and later education and work are platforms where many participants believe that they have been given space to grow and develop their own set of values. The participants were exposed to their original culture at home and other values outside the home, which influenced them. Several participants described that continuous adaptation to multiple value systems and arenas has been important for them and where they are now.

Completing higher education has been directional for Sara and stands out as something she has held onto during tough periods of her life. It has also been important for Sara’s value system:

The fact that I have taken higher education, I think that has had a very big impact. This is especially true in regard to self-definition and understanding. To see things from different perspectives, ask self-critical questions to oneself… or to be exploratory.

Marlon described becoming adaptable after having to find his place in Norway on his own, which has been an advantage in his working life. Marlon has combined three jobs simultaneously to create a safe platform for the family to grow.

I think I shouldn’t judge anyone; I think that whether you are a child or a youth or an adult” (…) “you can stick to your culture, you can do that, but you must also adapt to the culture you have come to.

Language competence is important in several of the participants’ lives. Although it has been difficult to find their place in Norway and get in touch with Norwegians, both Amina and Leyla experience that language has contributed to their feeling that their multicultural background is useful in Norway. Leyla reports that she speaks five languages fluently. Learning languages at an early age has contributed to her in that it can help her with communication in the workplace or in situations where she has helped her own family obtain health care.

All the participants came from very different cultures but described the significance of similar attitudes in meeting Norwegian culture. They particularly emphasize that their experience of standing with one foot in each culture has promoted the ability to adapt and given an open and exploratory approach to values.

## Discussion

The participants’ life stories show how formative encounters in Norway mold their self-perception and sense of belonging through experiences that intersect feelings of strangeness and opportunity. These experiences are organized through three central themes: the sense of “outsiderness” in both the new culture and the culture of origin, the conflicts and reconciliations of values, and the unique position of navigating between two cultures. This triangulation of themes highlights the complexity of acculturation processes and the accompanying psychological and social struggles.

The first theme, “Experiences of being a stranger: Outsiderness among those I came to and those I left behind,” illustrates how refugees experience a strong sense of dislocation, not only in the host society but also in terms of their own cultural background. This dual alienation can increase the feeling of not fully belonging anywhere, resonating with the acculturation framework discussed by Berry [17], which posits that integration involves both maintaining aspects of one’s original culture and adopting elements of the new culture. However, as Schwartz et al. [19] emphasize, this process can be psychologically taxing, as individuals must constantly navigate between conflicting cultural expectations.

The second theme, “There will be value conflicts, but value reconciliation is possible,” delves into the tension between the collectivist values of the refugees’ countries of origin and the individualistic values of Norwegian society. This tension creates emotional and social challenges for the participants. Kuo [22] highlights the importance of coping mechanisms during acculturation, which may help individuals reconcile conflicting values. The study participants illustrate how, despite the difficulties, this reconciliation process can lead to personal growth and a deeper understanding of themselves, aligning with the concept of redemption themes described by McAdams and Bowman [43]. However, the psychological toll is significant, and for some, the process involves deep pain, especially in relation to family and cultural ties.

In the final theme, “A unique perspective: Navigating between two cultures,” the participants expressed that although they experienced considerable internal conflict, their bicultural existence also presented opportunities. This dual perspective allows them to gain unique insights into both cultures and, as McAdams and Bowman [43] described, can be a source of narrative identity that fosters resilience. However, as the study shows, this position often comes with the psychological burden of marginalization, which echoes the findings of Cheung et al. [20], who noted that younger immigrants tend to acculturate faster but may also face greater identity dissonance. The participants often feel as though they are “cultural tightrope walkers,” a metaphor that seeks to capture the existential tension they endure.

The life stories reveal the challenges young refugees face in creating coherence and unity in their narratives after arriving in Norway. While the experience of disruption and discontinuity associated with fleeing may not be as pronounced for young refugees as it is for adults, it remains a significant factor, particularly for our older participants. The more substantial challenge, however, lies in dealing with conflicting value systems and determining which principles should guide one’s actions and decisions. For some individuals, these value conflicts have led to deeply painful experiences, especially in relation to their family and cultural heritage.

The varying ages at which participants arrived in Norway may have played a role in shaping their experiences and how they interpret them over time. As Phoenix and Orellana [44] highlight; individuals reinterpret their past in light of their evolving life trajectories. They demonstrate how memories of childhood language brokering, for instance, are actively reshaped rather than merely recalled. Orellana and Phoenix [45] argue that re-interpretations of childhood experiences are not static but evolve alongside life events, social roles, and shifting socio-political contexts.

The participants who arrived at a young age. like Samira, Leyla and Razm, experienced migration as part of their formative years. Arriving before adolescence meant that they likely underwent much of their identity formation within the Norwegian context. This could potentially make their sense of belonging stronger. They had more time to integrate both linguistically and culturally. However, we see that they also struggled with a sense of “in-betweenness”, neither fully Norwegian nor fully tied to their country of origin.

Those who arrived as teenagers, such as at age 17, had already developed a strong sense of identity in their country of origin. Participants like Nora, Marlon, Amina, and Sara experienced migration at a particularly sensitive life stage—adolescence. At this age, they would have already developed a strong sense of identity, language skills, and cultural attachments from their home country. The transition might have been more of a challenge in itself. They reflect more strongly on their migration as a moment of rupture, as adolescence is already a time of identity formation, and moving countries compounded that experience. On the other hand, now, as adults, they tend to see their teenage adaptation struggles as defining moments. These experiences might also have shaped their resilience and independence.

Long versus short time since arrival might also have significance. Participants who have lived in Norway for two or three decades, such as Vince and Sara reflect on their migration experience differently than those who have been in Norway for only 13–15 years. They have more of a biographical overview. They see their migration as a central part of their life story, influencing career, family life, and social integration. They have experienced, and even taken part, in major socio-political changes in Norway, such as shifting attitudes toward immigration. Distance in time makes it possible for Vince to reinterpret past struggles with a sense of accomplishment, particularly as he now feels fully integrated. Both Sara and Vince also have had the possibility to experience generational shifts, such as raising children in Norway and negotiating their cultural heritage in a new way.

The participants’ self-narratives emerge from a particular field of tension between historically conditioned vulnerabilities related to flight and migration and existential vulnerabilities that this flight and migration process actualizes (cf. 4).

These life stories are not neatly tied to the “master plots” of either the culture of origin or the Norwegian culture, as McLean and Syed [46] term them, but are situated in a space of ambiguity and fluidity. The challenge of constructing a coherent narrative identity under these conditions is considerable. Participants’ self-narratives often reflect a struggle against certain forms of relational mirroring in both cultures, where they are defined in ways that they find limiting or painful.

The notion of existential anxiety, as an inevitable component of human life, becomes particularly salient in these narratives. The lack of “safe ground” when making value-based decisions exacerbates this anxiety. To a greater extent than people who primarily find their belonging in a culture, the question of value choices is brought to the fore, and the experience of having a «safe ground” under their feet when choosing is lacking, highlighting an existential anxiety that is at the same time an inevitable component of living a human life. This, in turn, will reveal emotional pain and an experience of constantly having to find balance as a cultural tightrope walker. As Giddens [6] argues, in late modernity, identity is increasingly reflexive and contingent, a dynamic particularly heightened in migration. The participants’ narratives reveal how they continually seek a balance between cultures, often finding themselves without clear footing in either, which underscores the emotional and psychological strain of acculturation.

The findings contribute to the broader discourse on refugee adaptation by highlighting not only the psychological and cultural challenges but also the potential for growth and mastery through narrative identity development. As Ward and Geeraert [18] suggested, acculturation processes are deeply embedded in ecological contexts, and this study shows how personal narratives are shaped by the interplay of individual experiences and broader cultural frameworks. The participants’ stories suggest that while the process of acculturation is fraught with challenges, it also opens up space for new forms of self-understanding and resilience.

### Reflexivity, scope and limitations

The researchers all have backgrounds from the Norwegian majority population, which may have resulted in the Norwegian cultural voice of the participants being more prominently expressed, both in interviews and data interpretation. However, throughout the interviews and analysis, we attempted to use this commonality as a shared platform to explore and invite aspects of participants’ experiences that were not as clearly rooted in Norwegian culture.

Values negotiation requires specific conditions to succeed. The freedom to choose values is itself a value—one that may, at times, be in tension with other core values. Traditional, collectivistic cultures often embed values that resist negotiation. There is an inherent challenge in interpreting these narratives: while participants expressed a sense of agency in reconciling different value systems, the very ability to engage in such negotiation reflects an implicit prioritization of individual autonomy. As researchers, we recognize that our analysis is not value neutral. We are shaped by an ontological and epistemological framework that takes for granted the desirability of personal freedom, equity, and self-determination. Our participants largely shared this perspective. However, it remains important to acknowledge that for some individuals and communities, the act of values negotiation itself may be seen as a compromise or even a loss. Even in cases like this, where there is broad agreement on the benefits of values negotiation, we must remain attentive to the sociopolitical contexts that make such negotiation possible. We must also be attentive to how dominant cultural narratives may shape both the participants’ experiences and our interpretation of them.

These life history interviews were conducted with participants recruited through organizations that tend to engage well-integrated immigrants, largely from middle-class backgrounds. In relation to Steinar Kvale’s [42] criterion of “transferability” in qualitative research, the findings may be somewhat limited to groups of people with refugee backgrounds who exhibit a relatively high degree of affiliation with Norwegian culture. Future studies on narrative identity formation at the intersection of two cultures could benefit from broader recruitment strategies and a larger number of participants, allowing for comparisons between groups with different socioeconomic backgrounds.

## Conclusion

This study highlights how young refugees in Norway have to orient themselves in complex cultural and psychological landscapes, where the experience of strangeness coexists with the potential for opportunity. These narratives underscore the need to study the lived experience of acculturation, one exploration of the psychological costs but also the possibilities for growth. This study contributes to our understanding of the refugee experience, emphasizing the importance of support systems that acknowledge both the pain and potential inherent in the acculturation process.

## Data Availability

The data that support the findings of this study consist of confidential life story interviews. Owing to ethical and privacy considerations, these data are not publicly available. The participants were assured of confidentiality, and access to raw data was restricted to protect their privacy.
